# Effect of educational intervention programme on the health-related quality of life (HRQOL) of individuals with type 2 diabetes mellitus in South-East, Nigeria

**DOI:** 10.1186/s12902-023-01329-y

**Published:** 2023-04-07

**Authors:** Christiana Nkiru Okafor, Christopher Olusanjo Akosile, Chiejina Edith Nkechi, Uchenna Prosper Okonkwo, Chinenye Mercy Nwankwo, Ijeoma Lewechi Okoronkwo, Pat Uzo Okpala, Anulika Johnson Afonne

**Affiliations:** 1 Department of Nursing Science, Faculty of Health Sciences and Technology, Nnamdi Azikiwe University, Awka, Nigeria; 2 Department of Medical Rehabilitation, Faculty of Health Sciences and Technology, Nnamdi Azikiwe University, Awka, Nnewi, PMB 5001 Anambra State Nigeria; 3 Department of Community Health, Kabela University School of Medicine, Kabale University, Kabale, Uganda; 4 Department of Nursing Sciences, Faculty of Health Sciences and Technology, University of Nigeria, Nsukka, Nigeria; 5 Department of Nursing, College of Health Sciences, Evangel University, Akaeze, Ebonyi State Nigeria

**Keywords:** Type 2 diabetes, T2DM, HRQOL, Educational intervention, Self-management education, Quasi-experimental

## Abstract

**Background:**

Diabetes is one of the most important chronic diseases that have a great impact on health as people with diabetes are constantly being reminded of their disease daily; they have to eat carefully, exercise, and test their blood glucose. They often feel challenged by their disease because of its day-to-day management demands and these affect their quality of life. The study aimed at determining the effect of an educational intervention program on the quality of life of Individuals with type 2 Diabetes Mellitus in South East, Nigeria.

**Methods:**

A quasi-experimental controlled study involving three hundred and eighty-two (382) type 2 DM persons recruited from the tertiary health institutions in South East, Nigeria, and randomly assigned to intervention and control groups respectively. Data was collected from the diabetic clinics of the health institutions using the SF – 36 questionnaires. Pretest data collection was done, and thereafter, education on self-care was given to the intervention group. After a 6months follow-up, post-test data were collected from both groups. Analysis was done using an Independent t-test, Analysis of Covariance (ANCOVA), Paired Samples Test, and Spearman rank order correlation at 0.05 alpha level.

**Results:**

The control group indicated significantly higher mean HRQOL scores in most domains of the HRQOL before intervention (t = -1.927 to -6.072, p < 0.05). However, 6 months after the intervention, the mean HRQOL scores of the intervention group increased significantly in all the domains of HRQOL (p < 0.05) with an effect size of 0.14 (Eta squared). A comparison of the two groups shows a statistically significant difference (64.72 ± 10.96 vs. 58.85 ± 15.23; t = 4.349. p = 0.001) after the intervention. Age was inversely correlated with some domains of HRQOL; as age increases, HRQOL decreases in those domains. Gender had no significant influence on HRQOL.

**Conclusion:**

Educational intervention was effective in improving HRQOL in individuals with type 2 DM. Hence, it is recommended for inclusion in all diabetes care plans.

**Supplementary Information:**

The online version contains supplementary material available at 10.1186/s12902-023-01329-y.

## Introduction

Diabetes Mellitus (DM) is a metabolic disorder known to affect people of all ages and racial backgrounds. It has been acclaimed as one of the major health challenges ravaging the global community [1]. In the past, it was known to affect the affluent more than the non-affluent but in the contemporary periods, its effect is being felt significantly in developing countries [2]. The previous report has shown that as high as 80% of diabetes-related deaths were recorded in low and middle-income countries [1].

Interestingly, previous studies have reported a progressive increase in the prevalence of diabetes both at global, regional, and national levels [1, 3–5]. In 2011, it was estimated that 285 million adults were affected with DM globally [4]. Also, in 2013, another report had that about 382 million adults globally were affected by DM with a prevalence of 3.8%. In 2014, the global prevalence rose to 9% with an estimated 387 million adults living with diabetes [5]. A report from a previous study revealed that nearly half a billion adults globally were estimated to be living with diabetes [6]. A previous study reported the prevalence of diabetes in Nigeria to be within the region of 8 − 10% with over 4 million cases as reported by another study [4, 7]. Because of the rising global prevalence of diabetes associated with poor quality of life, the WHO projected that diabetes may become the 7th leading cause of death by 2030 [8].

It has been posited that diabetes mellitus often results in frequent hospital hospitalization which is associated with high economic costs, and consequently affects the quality of life of persons with diabetes [9]. Hence, attention to strategies such as patient training and education to promote quality of life is critical to reducing early complications and readmissions of patients with chronic diseases such as diabetes.

The QOL represents the effect of illness on a person as perceived by the person [1, 4]. Quality of life also encompasses people’s emotional, social and physical well-being and their ability to function in the ordinary [10]. From the perceptive of the WHO, the QOL of an individual is perceived as their position in life in the context of the culture and value system in which they live and about their goal, expectations, standards, and concerns [11]. The perception of the meaning of QOL varies from one individual to another, and from one group to another group. These differences in the definition stem from the multi-disciplinary use of the term. Hence, the Centers for Disease Control (CDC) explained QOL to mean a broad multidimensional concept that usually includes subjective evaluation of both positive and negative aspects of life [12]. However, HRQOL is put in context when the QOL is considered concerning the impact on Health and Disease [12]. Health-Related Quality of Life is a health outcome that quantifies how disease, disability, or disorder affects an individual’s well-being. [12, 13]. According to the WHO, HRQL is measured in the dimensions of the physical, mental, and social well-being of an individual [14].

Diabetes is one of the most important chronic diseases that have a great impact on health. People with diabetes are constantly being reminded of their disease daily; they have to choose their diet as well as decide when to schedule their meals, they also have to exercise, test their blood glucose, take their medication, monitor their blood pressure, check for symptoms of hyper or hypoglycemia as well as deal with the fear of the possibility of complications. As a result, they often feel challenged by their disease because of its day-to-day management demands and these may affect their quality of life. Diabetes education is concerned with encouraging independence and self-confidence so that people carry out their self-care activities. Knowledge of self-management of diabetes is an important aspect of better glycemic control and better quality of life. The aim is to enable the patient to become the most knowledgeable and hopefully the most active participant in his or her diabetes care [15]. It also aims at optimizing metabolic control, preventing acute and chronic complications, and improving quality of life [16]. However, previous studies on self-care practice revealed that persons with DM have inadequate knowledge of self-care [17, 18]. This, the researchers assumed may affect their HRQOL. We realized that there was a dearth of studies on non-pharmacological interventions in the care of people with type 2 diabetes in Nigeria, especially in the South Eastern part. This has therefore created a knowledge gap that needs to be filled hence our desire to find an answer to the research question, what will be the effect of an educational intervention program on the health-related quality of life (HRQol) of individuals with type 2 diabetes mellitus in South-East, Nigeria? We hereby hypothesized that an educational intervention program on the HRQOL of individuals with type 2 DM recruited from selected tertiary institutions in South East, Nigeria will not lead to an improvement in their HRQOL. The success of the incorporation of educational intervention in the management of type 2 DM will go a long way in improving the HRQOL of individuals with type 2 diabetes.

## Methodology

### Study type

The study was a multi-center quasi-experimental design involving three hundred and eighty-two (382) persons living with type 2 DM purposively recruited from the diabetic clinics of four tertiary health institutions in South East, Nigeria. Ethics approval to carry out the research was obtained from the Institutional Ethics Committee of Nnamdi Azikiwe Teaching Hospital University, University of Nigeria Teaching Hospital, and Federal Medical Center, Umuahia.

**Step 1. Selection of Study Area/States used for the study -** There are five (5) States that make up the South Eastern Region of Nigeria. Each State houses two tertiary health institutions making a total of ten (10) tertiary health institutions in South Eastern, Nigeria. These States with their tertiary health institutions were listed and a simple random technique with replacement was used to select four (4) States with their tertiary health institutions. The technique involved writing the name of each state on a piece of paper, folded and placed in a bag, a child was asked to pick from the bag one piece of paper at a time. The state picked was written down, and the piece of paper was folded and put back in the bag. This procedure was repeated until four States were selected. The States selected were: Abia, Anambra, Enugu, and Imo States.

**Step 2: Selection of Study center/ site –** Simple random technique was used to select a health institution from each state that making a total of four (4) tertiary health institutions that were used for the study. The institutions are Federal Medical Center, Umuahia, (FMCU), Nnamdi Azikiwe University Teaching Hospital, Nnewi, (NAUTHN), University of Nigeria Teaching Hospital, Ituku-Ozalla, (UNTHI), and Federal Medical Center, Owerri (FMCO).

**Step 3: Determination of Experimental (Intervention) and Comparison (Control) groups –** Participating tertiary health institutions were assigned to experimental and control groups by randomly assigning each to experimental and comparison (control) health institutions using simple randomization with replacement. This was achieved by writing the number 1,2,3,4,5,6 on a piece of paper, folded, and placed in a tray. Four girls, (each representing a health institution) were asked to pick a piece of paper from the tray. Odd numbers formed experimental hospitals while even numbers form comparison (control) hospitals. The institutions picked for experimental were UNTH Ituku-Ozalla and FMC Owerri, whereas NAUTH Nnewi and FMC Umuahia were picked as control hospitals. Hence, participants from UNTH Ituku-Ozalla and FMC Owerri formed the experimental (intervention) group while those from NAUTH Nnewi and FMC Umuahia formed the control (comparison) group. The Original sample population for the study was 410. A proportionate sampling technique was used to determine the number of participants recruited from each study site based on the proportion of people living with diabetes mellitus (PLWDM) from each site to the entire population of PLDM from the 4 hospitals selected for the study. Thus, experimental hospitals which were UNTH Enugu and FMC Owerri had 121 & 86 respectively. People living with diabetes respectively, total = 207. Control hospitals which were NAUTH Nnewi and FMC, Owerri had 103 & 100 PLWDM respectively, making a total of 203 PLWDM for the control group. However, before the intervention, it was observed that some copies of the questionnaire (9 from the experimental & 10 from the control group) were not properly filled/completed. Also, during the post-test, 9 participants from the control group did not show up, as a result, their pretest scores were removed. In total, we recorded an attrition of 28 out of the 410. So the analysis of the questionnaire was based on 198 experimental participants’ scores and 184 control participants’ scores.

### Step 4

Finally, a purposive sampling technique was used in recruiting participants for the study. The researcher met the diabetic persons at the diabetic clinics in the selected health institutions (Experimental and Control health institutions) on different occasions, after introducing the purpose of the study and the steps/procedures involved to them, those that opted for the study and who met the inclusion criteria were recruited. Their names and phone numbers or support persons’ phone contact were collected.

**Patient Education-** Educational intervention covered areas such as meaning, types, causes, complications of DM, adherence to diet therapy, blood glucose monitoring, physical activity/exercise, foot care, adherence to medication, recognition of symptoms of hypo and hyperglycemia, and actions to take, blood pressure monitoring, regular health checkups on eye care, health care use, 3-monthly laboratory test for glycosylated hemoglobin (HbAic), communication with the physician, lifestyle changes, managing emotional problems as well as stress management.

### Method of data collection

#### Research Assistants

Six research assistants (final year student nurses) trained by the researchers assisted in data collection from the selected health institutions. All the research assistants received training on the areas they were to assist in the study. Each item in the questionnaire was explained to them and the need to maintain objectivity was emphasized. The training of research assistants lasted for two weeks.

The study participants were shared into groups of not more than 25 persons/group for easy administration of the questionnaire as well as education of intervention group participants. Each group of study participants was invited to the clinic on a particular day in the week for pre-intervention data collection. Pretest data were collected from study participants (both experimental and control group) who met the inclusion criteria, using the English version of the questionnaire. However, a specialist in the native language (Igbo language), who was earlier trained on the purpose of the study, was involved in translating the questionnaire for non-literate participants. Pretest data collection lasted for 6weeks.

#### Educational intervention

Educational intervention material centered on general diabetes management such as involvement in physical activity/exercise, diet adherence, foot care, monitoring of blood sugar, blood pressure monitoring, recognition of signs of hypo and hyperglycemia and actions to take, and eye checkups. Other areas covered include lifestyle changes (avoidance of intake of alcohol/sweetened wine, cigarette smoking, etc.), involvement in healthy social functions (joining the diabetic club, etc.) health care use (even in the absence of symptoms), communication with physician, lifestyle changes, emotional and stress management.

The diabetes self-management education commenced for the experimental group and lasted for 9 weeks. An unpublished booklet titled **“**Managing Your Diabetes**”** developed by the researchers from a module on diabetes education and other relevant materials was given to the experimental group to go home with. The experimental group was followed up, two weekly meetings were arranged with them to emphasize more on diabetes self-management and also encouraged them to practice self-management. Phone calls were made between meetings to answer the participant’s questions. Also, the two weekly meetings helped the researchers to be having contact with the experimental participants to identify the areas they were having a problem with the practice of self-care. The control group participants received normal care during the period of intervention. After six months of commencement of training with follow-up, copies of the questionnaire on quality of life were administered as a posttest to both the experimental and control groups to observe the effect of the education on the quality of life of the intervention (experimental) group.

At the end of the post-test data collection activities, the researchers educated the participants in the control group and gave each of them a copy of the educational material as means of support. The educational materials were leaflets that contain brief but catchy/vital information on diabetes e.g. causes, prevention, and medical treatment for diabetes. Both groups and their family members/caregivers were given psychoeducation as part of the measures to help them accept the condition in which they found themselves and to assist the loved ones comply with the instructions given during educational interventions. Psychoeducation includes information on how to explain aspects of living with an illness to family members so that they can understand the effect of the illness and assist the patient and treatment providers in the treatment program. There is evidence that psychoeducation improves the outcomes of mental illness and many other medical illnesses [19, 20].

**An instrument for data collection**: Data was collected using the Rand Short Form 36 (SF-36) Health Survey. SF-36 questionnaire has a total of 36 questions with eight (8) scales that measure 8 dimensions (domains) of an individual’s health. Each scale contains specific questions that assess the quality of life in that domain. The domains are: Physical functioning contains ten (10) questions, Role limitation due to physical health (4 questions), Role limitation due to emotional problem (3 questions), Energy/Fatigue (4 questions), Emotional well-being (5 questions), Social functioning (2 questions), Pain (2 questions) and General health (6 questions). The SF-36 has been validated for use in Nigeria population by two previous studies; the first study on sickle cell disease patients attending outpatient clinics in Ibadan, reported that the reliability of each of the dimensions was above 0.70. Item internal consistency ranged from 0.42 to 0.91 and scaling success ranged between 0.98 and 100% [21], while the second study on translation, cross-cultural adaptation and psychometric evaluation of Yoruba version of the short-form 36 health survey reported that the concurrent validity of the Yoruba SF-36 was high, with scales and domains having co-efficient ranges greater than 0.70 that was considered desirable for good validity of a new tool. Also, the convergent validity was satisfactory, ranging from 0.421 to 0.907 [22]. Similarly, in the current study, SF-36 was tested relative to the current sample before application, and it shows acceptable internal consistency (0.63– 0.95), known-group validity (0.60–0.99), convergent validity, and ceiling and floor effects.

The above domains of the HRQOL have further grouped into two components viz.: the physical and the mental components. Scoring of SF – 36 questionnaires was done using RAND Scoring guide. All questions were scored on a scale from 0 to 100, with 100 representing the highest level of functioning possible. Aggregate scores were compiled as a percentage of the total points possible, using the RAND scoring table. The scores from those questions that addressed each specific area of functional health status were averaged together, for a final score within each of the dimensions measured. The scores were entered into SPSS for statistical analysis.

### Method of data analyses

The data were analyzed using IBM Statistical Packages for the Social Sciences (SPSS 25.0: SPSS Inc., Chicago, IL, USA). The Socio-demographic characteristics data were summarized using descriptive statistics of frequency count, percentages, mean, and standard deviation. An Independent t-test was used to compare the baseline QOL scores between the experimental and control groups before the intervention. Analysis of Covariance (ANCOVA) test statistics was used to compare the changes that occurred in HRQOL between the experimental and control groups 6 months’ post-intervention. Paired samples test was used to examine the changes that occurred between the components of HRQOL. Spearman rank order correlation was used to test the relationship between age and HRQOL domains. T-test was used to test the association between gender and the domains of the HRQOL in all tests, p-value less than 0.05 alpha levels were considered significant.

## Results

Table 1 shows that the comparison of the number of both male and female participants was matched. They were not statistically significant p = > 0.256.

**Table 1:** Demographic Characteristics of study participants

Characteristics	GROUP			χ^2^	P-value
	Exp. Freq.	Control Freq.	Total(%) Freq.		
**Gender**					
Male	79 (39.9)	84 (45.7)	163 (42.7)	1.290	0.256
Female	119 (60.1)	100 (54.3)	219 (57.3)		
**Total**	**198 (51.8)**	**184 (48.2)**	**382 (100%)**		

Table 2 shows that both groups had similar proportions of participants across gender. No significant difference was observed between the groups regarding gender. The mean age of participants in the experimental group **(58.52 ± 11.40, t = 1.87)** was similar to that of the control group **(56.29 ± 11.92, t = 1.86, p = 0.063).**

**Table 2:** Mean Age of Study Participants

Characteristic	Group	N	Mean	SD	T-test	P–value
**Age**	**Exp.**	198	58.52	11.40	1.87	0.063
	Control	184	56.29	11.92	1.86	

Table 3: shows the mean and standard deviation of quality of life scores of the experimental and control groups before educational intervention. Independent t-test result shows significantly higher mean QOL scores in the control group before intervention in the following domains: Energy/fatigue **(57.03 ± 17.20 vs. 51.60 ± 14.15; t = -3.379, p = 0.001)**, Emotional wellbeing **(67.64 ± 16.02 vs. 59.19 ± 12.96; t = -5.690, p = 0.001)**, Social functioning **(62.96 ± 21.37 vs. 58.33 ± 20.09; -2.187, p = 0.029)**, and General Health **(56.95 ± 16.66 vs. 47.75 ± 12.85; t = -6.072, p = 0.001)**. The table further shows differences in overall QOL between the groups; the control group had a significantly higher overall QOL mean score than the experimental group before intervention **(56.51 ± 16.44 vs. 52.02 ± 15.02, t = -2.792, p = 0.006).**

**Table 3:** Independent t-test comparison of Health-Related Quality of Life (HRQOL) scores between Experimental and Control groups before intervention

QOL Domains	Group.	Mean	SD	t-test	p
**Physical Functioning**	Exp.	56.49	24.50	0.674	0.501
	Cont.	54.86	22.53		
**Role physical**	Exp.	43.99	36.37	0.381	0.703
	Cont.	42.59	35.31		
**Role emotional**	Exp.	47.48	36.76	–1.762	0.079
	Cont.	54.17	37.58		
**Energy/fatigue**	Exp.	51.60	14.15	–3.379	0.001*
	Cont.	57.03	17.20		
**Emotional Wellbeing**	Exp.	59.19	12.96	–5.690	0.001*
	Cont.	67.64	16.02		
**Social functioning**	Exp.	58.33	20.09	–2.186	0.029*
	Cont.	62.96	21.37		
**Pain**	Exp.	51.33	22.09	–1.927	0.055
	Cont.	55.88	24.09		
**General Health**	Exp.	47.75	12.85	–6.072	0.001*
	Cont.	56.95	16.66		
**Overall QOL (before intervention)**	Exp.	52.02	15.02	–2.792	0.006
	Cont.	56.51	16.44		

Table 4 shows a comparison of quality of life scores between experimental and control groups before and after the educational intervention. Before the intervention, the control group had significantly higher QOL mean scores than the experimental group in the following QOL domains: energy/fatigue **(57.02 ± 17.20 vs.** 51.60 ± 14.15, **p = 0.001)**, emotional wellbeing **(67.64 ± 16.02 vs.** 59.19 ± 12.96, **p = 0.001)**, social functioning **(62.96 ± 21.37 vs.** 58.33 **±** 20.09, **p = 0.029)**, general health (**47.75 ± 12.85 v s56.95 ± 16.66, t = -6.072, p = 0.001**). However, 6-months after the intervention, the experimental group made significantly better improvements in all QOL domains compared to the control group (p < 0.05), Eta-squared (**η**^**2**^) = **0**.14. This is an indication that the educational intervention administered on the participants has a large effect size. The overall QOL mean score of the experimental group was observed to be significantly higher by 5.87 than the control group after the intervention (p = 0.001).

**Table 4:** ANCOVA table comparing changes in quality of life between experimental and control groups 6 months after intervention (Posttest)

QOL Domains		EXP	CONTROL	F-value	p-val.
		X ± SD	X ± SD		
**Physical functioning**	Pre-T	56.49 ± **24**.50	54.86 ± 22.53		
	Post-T	69.22 ± **18**.58	57.75 ± 18.59	4.491	0.001*
**Role limitation - physical**	Pre-t	43.99 ± 36.37	42.59 ± 35.31		
	Post-t	69.29 **±** 25.08	49.49 **±** 30.77	13.01	0.001*
**Role limitation - emotional**	Pre-t	47.48 ± 36.67	54.17 **±** 37.58		
	Post-t	75.43 ± 23.56	60.78 ± **32.83**	15.063	0.001*
**Energy/fatigue**	Pre-t	51.60 ± **14.15**	57.02 ± **17.20**		
	Post-t	56.47 **±** 11.37	57.55 ± 16.12	3.579	0.001*
**Emotional wellbeing**	Pre-t	59.19 ± 12.96	67.64 ± 16.02		
	Post-t	65.03 **±** 12.92	67.64 ± 16.02	3.895	0.001*
**Social Functioning**	Pre-t	58.33 **± 20**.09	62.96 ± **21.37**		
	Post-t	61.65 ± **17**.85	62.96 ± **21.37**	1.858	0.018*
**Pain**	Pre-t	51.33 ± **22**.09	55.88 ± **24.09**		
	Post-t	59.88 ± 15.97	56.93 ± **21.72**	2.770	0.001*
**General Health**	Pre-t	47.75 **±** 12.85	56.95 **±** 16.66		
	Post-t	60.78 ± 15.52	57.68 ± **16.27**	10.194	0.001*
**Overall QOL (6 months after intervention)**	Pre-t	52.02 ± 15.02	56.51 ± 16.44		
	Post-t	64.72 ± 10.96	58.85 ± 15.23	4.349	0.001*

Eta-squared (**η**^**2**^) = **0** 0.14.

Table 5 further showed significant differences in the QOL mean score of the physical and mental components 6 months’ post-intervention. The physical component QOL mean increased by 9.12points higher than the pretest QOL mean, while the mental component QOL mean increased by 6.3points higher than the pretest QOL mean, thus indicating the effectiveness of the intervention on both components of QOL although the effect was more on the physical component **(t = -14.51)** than the mental component **(t = -10.82).**

**Table 5:** Comparison of pre-test and post-test of all participants’ physical and mental QOL components summaries using Paired Samples test

Components of QOL	Pretest	Posttest	t-test	P–val
	Mean ± SD	Mean ± SD		
**Physical Component**	51.18 ± 17.98	60.30 ± 16.42	–14.51	0.001
**Mental component**	57.18 ± 16.79	63.48 ± 13.60	–10.82	0.001

• **Cd – Correlation of difference**

Table 6 shows an inverse correlation between age and the following domains of HRQOL: Physical functioning **(γ = − 0.175, p = 0.001**), role limitation due to physical health **(γ = − 0.219, p = 0.001)**, energy/fatigue **(γ = − 0.102, P = 0.047)**, Pain **(γ = − 0.117, P = 0.022)**. As age increases, QOL decreases in the above-mentioned domains.

**Table 6:** Spearman rank order test showing a correlation between age and the individual domains of the Health-Related Quality of Life of individuals with type 2 DM

Quality of Life domain	R	C.d	P val.
**Physical functioning**	-0.175	0.031	0.001*
**Role limitation due to physical Health**	-0.219	0.048	0.001*
**Role limitation due to emotional health**	-.089	**0.0079**	0.082
**Energy/fatigue**	-0.102	0.010	0.047*
**Emotional wellbeing**	-0.027	0.0007	.600
**Social functioning**	-0.032	0.0010	.529
**Pain**	-0.117	0.014	0.022*
**General health**	-0.082	0.0067	.108

Table 7. shows no significant relationship between gender and QOL (p ˃ 0.05).

**Table 7:** T-test showing the relationship between gender and the individual domains of the Health-Related Quality of Life domains of individuals with T2DM

Quality of life domains	Gender	No.	Mean	SD	t-test	p-value
**Physical Functioning**	Male	163	54.10	24.09	–1.147	0.252
	Female	219	56.90	23.13		
**Role physical**	Male	163	43.15	36.80	-0.076	0.939
	Female	219	43.44	35.16		
**Role emotional**	Male	163	51.02	39.14	.145	0.886
	Female	219	50.42	35.80		
**Energy/fatigue**	Male	163	53.63	16.63	-0.617	0.537
	Female	219	54.65	15.37		
**Emotional wellbeing**	Male	163	62.49	15.96	-0.852	0.395
	Female	219	63.83	14.43		
**Social functioning**	Male	163	61.44	20.25	.710	0.478
	Female	219	59.91	21.26		
**Pain**	Male	163	54.03	22.26	.368	.0.713
	Female	219	53.14	23.85		
**General Health**	Male	163	51.79	15.28	-0.418	0.676
	Female	219	52.47	15.67		

## Discussion

The baseline findings on HRQOL revealed that a good number of the study participants scored above 50 in most domains of QOL at the pretest except in role limitation due to physical health in which more than half of all participants scored below 50. This finding concurs with a previous finding which reported QOL scores of more than 50 in most domains of the SF – 36 measurements [23]. HRQOL between the two groups before intervention showed fewer proportions of participants in the experimental group scored above 50 in most domains of the SF – 36. Independent t-tests on baseline QOL showed that both groups were similar except in the domains of energy/fatigue, emotional well-being, social functioning, and general health where the control group indicated a significantly higher mean QOL score. This implies that control group participants experience less fatigue, and have better emotional well-being, better social functioning, and general health than the experimental group before intervention. This finding disagrees with the findings of a previous study that reported poor QOL in all the domains of QOL in both experimental and control groups before their educational intervention [24].

A significant difference was also observed in the overall mean QOL score of the intervention and control groups at the pretest stage, the control group had a higher overall mean QOL score than the intervention group. This further showed that the control group participants had a better QOL than the intervention group before the intervention.

However, 6 months after educational intervention, the mean QOL scores of the experimental group increased significantly in all the domains of QOL. Also, the overall mean QOL score of the intervention group increased significantly by 5.87 points after the intervention. This implies a positive effect of the intervention on the experimental group as shown in the HRQoL scores. This underscores the fact that educational intervention for people living with diabetes will be helpful in the non-medical management of DM. The outcome of the study runs counter to the study hypothesis that stated that an educational intervention program on the HRQOL of individuals with type 2 DM recruited from selected tertiary institutions in South East, Nigeria will not lead to an improvement in their HRQOL. This finding is similar to the findings of the previous finding in Saudi Arabia which revealed statistically significant improvement in four dimensions of HRQOL after their psychoeducational intervention (P < 0.01) [25]. It is also similar to the finding of another study in Iran, which revealed a significant difference in mean scores of physical, psychological, and social domains of QOL after the intervention [26].

Age was inversely correlated with the domains of physical functioning, role physical, energy/fatigue, and pain. This implies that as age increases, QOL decreases in these domains. Several studies found that quality of life (QOL) in their study population worsens with increasing age [27–31]. This may be due to a high rate of comorbidities and other health challenges associated with old age. Also, the finding on age with pain is similar to the finding of a study that reported that participants over 60 years in their study experience bodily pain [23]. Further, the association between age with role limitation also agrees with the findings of a study where the age of their participants influenced QOL in the dimensions of role limitation and physical endurance [32]. In this study, gender had no significant association with any of the domains of HRQOL. This implies that being a male or a female does not produce any difference in the participants’ response to HRQOL scores after the educational intervention. We speculate that what matters most is the patient’s compliance with the diabetic educational management instructions given to the participants more than their gender. The HRQol scores of the participants are not influenced by the participants being males or females rather they could be influenced by the mastery of the standard management plans. This contradicts the findings of Miguel, et al. (2014) in which significant differences were observed between men and women in the domains of pain and social functioning (P < 0.05) [25]. It also contradicts the findings of Mahmoud et al. (2016) that revealed male participants to be better than female participants in HRQOL (P < 0.05) [32].

A significant difference was observed in the physical components (PCS) and mental components (MCS) QOL mean score after the intervention; the PCS increased significantly by 9.12points higher than the pretest mean, while the MCS increased by 6.3, thus indicating the effectiveness of the intervention on the PCS and MCS components of HRQOL. A similar study observed similar findings of an increase in QOL scores in PCS and MCS of their participants after 6 months of intervention (P < 0.05) [24].

## Conclusion

Educational intervention was effective in improving the quality of life in individuals living with type 2 diabetes mellitus with a large effect size (0.80). This is reflected in the significant improvement in the HRQOL of the experimental group after educational intervention as against the control group that did not receive the intervention but just relied on routine diabetic care. The large effect size shows that the educational intervention was very effective in improving the quality of life of those living with DM, and should be incorporated as an adjunct in the management of DM.

### Limitations of study

The quasi-experimental design used may limit the study’s ability to conclude a causal relationship between the educational intervention and the outcome. Also, the sample size was not adequate for a study of this magnitude hence generalisability of the outcome should be done with caution.

### Contribution to knowledge

This study was an attempt to find out the effect of an educational intervention program on the QOL of individuals living with type 2 DM in South East, Nigeria. We believe that the incorporation of educational intervention in the management of type 2 DM will go a long way in minimizing the development of comorbidities and drug intervention in diabetic patients hence improving the HRQOL of individuals with type 2 diabetes. The outcome has shown that when educational intervention is diligently delivered by the concerned health professionals and complied with by the diabetic patients that positive outcomes in HRQOL are guaranteed. There is a need for health managers to develop a policy that will encourage different health institutions and professionals to incorporate educational intervention in the management of type 2 diabetic patients in their practices. Self-management education should be included in the diabetes care plan and should be given serious attention. We recommend that future studies will be a randomized controlled study involving different regions of the country so that the cause-and-effect relationship will be determined.

## Electronic supplementary material

Below is the link to the electronic supplementary material.

Supplementary Material 1

## Data Availability

The data is with the corresponding author and will be made available at a reasonable request.

## Abbreviations

ANCOVA: Analysis of Covariance
QoL: Quality of life
T2DM: Type 2 diabetes
HRQOL: Health-related quality of life
DM: Diabetes Mellitus
WHO: World Health Organization
HbAic: Glycosylated hemoglobin
Cd: Correlation of difference
IDF: International Diabetes Federation
CDC: Center for Disease Control
NAUTH: Nnamdi Azikiwe University Teaching Hospital
UNTH: University of Nigeria Teaching Hospital
FMCO: Federal Medical Center Owerri
FMCU: Federal Medical Center Umuahia
PLWDM: People living with diabetes Mellitus
