# Supplementary material for: Effect of educational intervention programme on the health-related quality of life (HRQOL) of individuals with type 2 diabetes mellitus in South-East, Nigeria
Source: BMC Endocr Disord. 2023 Apr 7;23:75. doi: 10.1186/s12902-023-01329-y (PMC10080927; doi:10.1186/s12902-023-01329-y)
Supplement: Supplementary file 1 — Supplementary Material 1 [file 12902_2023_1329_MOESM1_ESM.docx]

**Appendix XII**

**Educational Intervention material**

**MANAGING YOUR DIABETES**

**BY OKAFOR CHRISTY NKIRU**

**Introduction**

Diabetes mellitus is a [chronic disease](file:///\\wiki\Chronic_disease), for which there is no known cure except in very specific situations. Management concentrates on keeping blood sugar levels as close to normal, without causing low blood sugar. This can usually be accomplished with a healthy diet, exercise, weight loss, and the use of appropriate medications (insulin in the case of type 1 diabetes; oral medications, and possibly insulin, in type 2 diabetes).

Learning about the disease and actively participating in the treatment is important since complications are far less common and less severe in people who have well-managed blood sugar levels. The goal of treatment is a glycosylated (HbA_1C)_ level of 6.5%, but should not be lower than that. Attention is also paid to other health problems that may accelerate the negative effects of diabetes. These include [smoking](file:///\\wiki\Tobacco_smoking), [elevated cholesterol](file:///\\wiki\Hypercholesterolemia) levels, [obesity](file:///\\wiki\Obesity), [high blood pressure](file:///\\wiki\Hypertension), and lack of regular [exercise](file:///\\wiki\Exercise). [Specialized footwear](file:///\\wiki\Orthotics) is widely used to reduce the risk of ulceration, or re-ulceration, in at-risk diabetic feet.

**Meaning of Diabetes mellitus (DM)**:

Diabetes Mellitus (DM) commonly referred to as **diabetes** is a group of metabolic diseases in which there are high blood sugar levels over a prolonged period.

**Types** include

- Type 1 Diabetes Mellitus: [Type 1 DM](file:///\\wiki\Diabetes_mellitus_type_1) results from the pancreas's failure to produce enough insulin. This form was previously referred to as "insulin-dependent diabetes mellitus" (IDDM) or "juvenile diabetes".
- Type 2 Diabetes mellitus was previously known as "non-insulin-dependent diabetes mellitus" (NIDDM) or "adult-onset diabetes". [Type 2 DM](file:///\\wiki\Diabetes_mellitus_type_2) begins with [insulin resistance](file:///\\wiki\Insulin_resistance), a condition in which cells fail to respond to insulin properly. As the disease progresses a lack of insulin may also develop. The primary cause is excessive body weight and not enough exercise.
- Gestational Diabetes Mellitus: [Gestational diabetes](file:///\\wiki\Gestational_diabetes), is the third main form and occurs when a pregnant woman without a previous history of diabetes develops high blood sugar levels. GDM resembles type 2 DM in several respects, involving a combination of relatively inadequate insulin secretion and responsiveness. It occurs in about 2–10% of all pregnancies and may improve or disappear after delivery. However, after pregnancy approximately 5–10% of women with gestational diabetes are found to have diabetes mellitus, most commonly type 2.
- Other types of DM

**Causes of DM –** This depends on the type

- The cause of type 1 DM is not fully known but may be linked with genetics, environmental, viral, and chemical/drugs causes
- The primary cause of type 2 DM is excessive body weight and not enough exercise

**Signs/Symptoms:** The major symptoms of high blood sugar are

- [Frequent urination](file:///\\wiki\Polyuria)
- [Increased thirst](file:///\\wiki\Polydipsia)
- [Increased hunger](file:///\\wiki\Polyphagia).

**Treatment:** Prevention and treatment involve

- Maintaining a [healthy diet](file:///\\wiki\Healthy_diet)
- [Physical exercise](file:///\\wiki\Physical_exercise)
- Maintaining a [normal body weight](file:///\\wiki\Normal_body_weight)
- Avoiding the use of [tobacco](file:///\\wiki\Tobacco).
- Control of [blood pressure](file:///\\wiki\Blood_pressure) and maintaining proper foot care are important for people with the disease.
- Management of type 1 DM is mostly by the use of [insulin](file:///\\wiki\Insulin) injection.
- Type 2 DM may be treated with oral medications with or without insulin.
- Insulin and some oral medications can cause [low blood sugar](file:///\\wiki\Hypoglycemia).
- [Weight loss surgery](file:///\\wiki\Bariatric_surgery) in those with [obesity](file:///\\wiki\Obesity) is sometimes an effective measure in those with type 2 DM.
- [Gestational diabetes](file:///\\wiki\Gestational_diabetes) usually resolves after the birth of the baby.

**Complications:** All forms of diabetes increase the risk of long-term complications. These typically develop after many years (10–20yrs) but may be the first symptom in those who have otherwise not received a diagnosis before that time. This implies that if DM is left untreated, it can cause many complications. Complications of diabetes may include:

- [Cardiovascular disease](file:///\\wiki\Cardiovascular_disease) for example hypertension
- [Stroke](file:///\\wiki\Stroke)
- Damage to the kidneys, known as [diabetic nephropathy](file:///\\wiki\Diabetic_nephropathy), can lead to tissue scarring, urine protein loss, and eventually [chronic kidney disease](file:///\\wiki\Chronic_kidney_disease), sometimes requiring [dialysis](file:///\\wiki\Dialysis) or [kidney transplant](file:///\\wiki\Kidney_transplant).
- Damage to the nerves of the body, known as [diabetic neuropathy](file:///\\wiki\Diabetic_neuropathy), is the most common complication of diabetes. The symptoms can include numbness, tingling, pain, and altered pain sensation, which can lead to damage to the skin.
- [D](file:///\\wiki\D)[iabetes-related foot problems](file:///\\wiki\Diabetic_foot) (such as [diabetic foot ulcers](file:///\\wiki\Diabetic_foot_ulcer)) may occur and can be difficult to treat, occasionally requiring [amputation](file:///\\wiki\Amputation).
- Painful [muscle wasting](file:///\\wiki\Muscle_wasting) and weakness resulting from [proximal diabetic neuropathy](file:///\\wiki\Proximal_diabetic_neuropathy).
- [Damage to the eyes](file:///\\wiki\Diabetic_retinopathy) (retinopathy) is caused by damage to the blood vessels in the retina of the eye. This could lead to gradual loss of vision and eventually blindness

**TIPS FOR SELF-MANAGEMENT OF DIABETES**

The following information will help you in your self-management of diabetes

**Exercise**

- You should exercise for 30 minutes every day. It could be walking around your compound or trekking some distance to your workplace OR
- Exercise continuously for 20 minutes at at-least 3times per week
- Check your blood pressure before and after any exercise

**Diet**

- Eat more fruits and vegetables, fewer carbohydrates, less fat, and high protein
- Avoid red meat such as cow meat as they contain bad fat
- Use more dry fish in cooking
- Eat white meat like chicken preferably native fowl (okuko Igbo) or old layers if you can afford it. Bush meat also is very nutritious if you can afford it as it does not contain bad fat
- Add a vegetable to your meals whether carbohydrate, protein
- Avoid fatty and fried foods such as fried ripped plantain, akara, etc
- Don’t add extra salt to your food after cooking
- If you are overweight, cut down the size of food you usually eat to help reduce the number of calories in your diet
- Eat fruits high in dietary fiber such as cucumber, garden egg
- Take either one stick of banana or one apple or one orange daily

**Medications**

- Take your drugs (insulin or oral drugs) as prescribed by your doctor
- If you are on insulin therapy, make sure your food is ready before taking insulin
- If you notice any side effects, please consult your doctor
- Carefully avoid indiscriminate use of drugs or drugs not prescribed by your doctor

**Hygiene**

- Bath with mild toilet soap and water at least 2 times daily.
- Clean your feet properly
- Open your toes and wash in-between

**Daily Foot care/maintenance**

- Avoid walking bare-footed
- Avoid the use of corn or callus removers
- Bath the foot with mild soap
- Clean the nails with a soft brush
- Avoid in-growing toe nail by cutting your nails when grown
- Cut your nails carefully to avoid giving yourself a wound
- Dry the feet with special attention using lamb’s wool or soft cotton cloth between the toes when web spaces are moist
- Use oil, lotion, or lanolin cream to avoid dryness
- Wear appropriate footwear
- Wear socks to absorb perspiration
- If you notice any soreness or wound on lower limbs or toes (no matter how small, whether painful or painless), please consult your doctor

**Blood Glucose Monitoring**

- Check your blood sugar level with glucometer equipment at least 2 times a day particularly if you are on insulin.
- You can also test your urine with clinitest tablet or clinistix if you don't have glucometer equipment, if the result is abnormal, go to the lab for a blood glucose test
- In addition to urine testing, also watch out for symptoms of high blood sugar or low blood sugar level
- Always keep in touch with your healthcare provider if you don't have equipment for home blood glucose monitoring particularly if you are on insulin treatment
- If you are on oral medication for diabetes, take your drugs as prescribed by your doctor and maintain your diet of low carbohydrate and fat (low-calorie diet)
- Always go for glycosylated hemoglobin every 3 months to check whether your diabetes has been controlled

**Eye care**

- Go for an eye check every 6 months
- When you observe blurring of vision or pain in the eye without elevation of blood sugar, please consult your healthcare provider

**Blood pressure monitoring**

- Check your blood pressure every two days if you have BP equipment (BP monitor) or at least once a week if you do not have the equipment to monitor your BP at home
- When you observe high readings (elevated BP) more than two times on different occasions, please consult your doctor

**Use of Health care**

- Always keep an appointment with your healthcare provider
- See your healthcare provider every 3 months for a check even in the absence of illness or symptoms
- Always consult specialists in diabetes management (Endocrinologists)
- Avoid using herbal medicines in place of drugs recommended by your doctor
- Avoid fallacies and misconceptions about your disease condition (Diabetes mellitus). Consult your healthcare provider or diabetes educator for clarification
- Consult your healthcare provider immediately if you observe any little soreness on your toe. It is not "enyi ure", it may be diabetes foot ulcer in its early stage
- Do not depend on "ogwu Igbo" (native or traditional medicine) or food supplements only for your diabetes management, it may lead to the early development of complications and death
- Consult your doctor before taking any food supplement or any other medicine outside the ones prescribed by him
- Avoid using over-the-counter drugs, that is drugs bought from chemist shops or unlicensed health personnel

**Lifestyle**

- Avoid intake of alcohol and sweetened wine
- Avoid cigarette smoking or smoke from cigarette smokers
- Quit smoking – consult a counselor on how to quit smoking
- Eat healthily – consult a dietitian for healthy diabetic diets
- Eat to live and don’t live to eat
- Drink enough water, not less than 2500ml in a day

**Emotional management**

- Always find something that makes you happy, and watch comedy films (e.g Osuofia, Nkoli nwa Nsukka, Ada Mbano, coming to America, etc)
- Always be in a lighter mood even when things are not going the way you want
- Avoid too much stress
- Relax and always be happy even when you are unable to maintain normal levels of blood sugar for some time. Remember that your creator (God) has not forgotten you and cannot forget you
- Have a chat with your healthcare provider anytime you are emotionally distressed
- Attend helpful social activities
- Join any diabetic club/association within your locality
- Pray to your God and always trust him to help and strengthen you

**Managing stress**

Life is full of challenges because life is dynamic. One experiences new challenges and opportunities every day. The challenges are diverse and in every place – home, workplace, business, church, etc. Some of these challenges present themselves as obstacles to accomplishing daily tasks or life goals. No one escapes it because it is part of life. Stress can be simply said to be any interference that disturbs a person's mental and physical well-being. Stress is not harmful if managed well. Below are some guidelines for the successful management of stress

- Always be yourself, adopt a new way of looking at life; that is forming your philosophy of life
- Remember that you are responsible for your own emotional and physical well-being
- Don’t allow people or your disease condition to determine your joy
- Always pray to God and trust Him to help you
- Have a positive perception (view) and attitude to life because a negative view of life can result in a high-stress level
- If you see yourself thinking negatively, force yourself to think about the positive aspect of your situation
- Ensure adequate sleep because sleep is essential for the successful management of stress and health maintenance. Ensure you have 7 – 8hrs nights of sleep
- Do your exercise as prescribed
- Do not depend on drugs (sleep pills) to be able to sleep and do not depend on drugs for stress reduction
- Be reasonably organized: Set short-term, intermittent goals for yourself, and avoid long-term goals for yourself
- Assign priority to things you want to accomplish each day, be realistic, and do not expect too much of yourself
- Learn to say no sometimes. Do not accept too many responsibilities. Know your limits and be assertive.
- Avoid executive stress; do not carry work from the office to home
- Always be yourself, avoid competition in whatever form
- Do not be a perfectionist because true perfection is unattainable
- Let go of the past. Do not dwell on your past mistakes. Learn from your experiences and try not to make the same mistakes again and continue with your life
- Live in the present and focus on the future.
- Take a break from reality by watching meaningful/educative films, TV, and videos, reading books, and listening to the radio. These provide a relaxing and enjoyable break from reality.
- Eat healthy diet
- Carry out the above recommendations on diabetes self-management religiously

**Signs of hypoglycemia (Low blood sugar level)**

- Sweating
- Weakness
- Agitation
- Dizziness

**What to do**

- Check your blood sugar level immediately
- Take a sweetened drink or take a cube of sugar if your blood sugar level is very low
- Look for something to eat
- Monitor your blood sugar level
- Have enough rest

**Signs of Hyperglycemia (High blood sugar level)**

- Excessive thirst (hunger for fluid intake)
- Excessive urination
- Dehydration evidenced by very dry skin
- Lethargy

**What to do**

- Drink enough water
- Check your blood sugar level
- Take your drug as prescribed
- Consult your doctor immediately

**Conclusion**

Life is sweet if only you can manage it well. Diabetes is not the worst disease one can suffer. Hence, you are better than many. Read this book very well and carry out the above instructions religiously and you will enjoy your life better.
